# Altered B-Cell Expansion and Maturation in Draining Mesenteric Lymph Nodes of Inflamed Gut in Crohn’s Disease

**DOI:** 10.1016/j.jcmgh.2023.12.006

**Published:** 2023-12-24

**Authors:** Sonja Kappel-Latif, Prasanti Kotagiri, Lukas Schlager, Gabor Schuld, Natalie Walterskirchen, Vanessa Schimek, Gavin Sewell, Carina Binder, Johanna Jobst, Supriya Murthy, Barbara Messner, Stefanie Dabsch, Arthur Kaser, Paul A. Lyons, Michael Bergmann, Anton Stift, Rudolf Oehler, Lukas W. Unger

**Affiliations:** Division of Visceral Surgery, Department of General Surgery, Medical University of Vienna, Vienna, Austria; Jeffrey Cheah Biomedical Centre, Cambridge Institute of Therapeutic Immunology and Infectious Disease, University of Cambridge, Cambridge, United Kingdom; Division of Visceral Surgery, Department of General Surgery, Medical University of Vienna, Vienna, Austria; Jeffrey Cheah Biomedical Centre, Cambridge Institute of Therapeutic Immunology and Infectious Disease, University of Cambridge, Cambridge, United Kingdom; Division of Gastroenterology and Hepatology, Department of Medicine, University of Cambridge, Cambridge, United Kingdom; Clinical Institute of Pathology, Medical University of Vienna, Vienna, Austria; Division of Visceral Surgery, Department of General Surgery, Medical University of Vienna, Vienna, Austria; Clinical Institute of Laboratory Medicine, Medical University of Vienna, Vienna, Austria; Cardiac Surgery Research Laboratory, Department of Cardiac Surgery, Medical University of Vienna, Vienna, Austria; Division of Gastroenterology and Hepatology, Department of Internal Medicine III, Medical University of Vienna, Vienna, Austria; Jeffrey Cheah Biomedical Centre, Cambridge Institute of Therapeutic Immunology and Infectious Disease, University of Cambridge, Cambridge, United Kingdom; Division of Gastroenterology and Hepatology, Department of Medicine, University of Cambridge, Cambridge, United Kingdom; Jeffrey Cheah Biomedical Centre, Cambridge Institute of Therapeutic Immunology and Infectious Disease, University of Cambridge, Cambridge, United Kingdom; Division of Visceral Surgery, Department of General Surgery, Medical University of Vienna, Vienna, Austria; Division of Visceral Surgery, Department of General Surgery, Medical University of Vienna, Vienna, Austria; Jeffrey Cheah Biomedical Centre, Cambridge Institute of Therapeutic Immunology and Infectious Disease, University of Cambridge, Cambridge, United Kingdom

**Figure 1:**
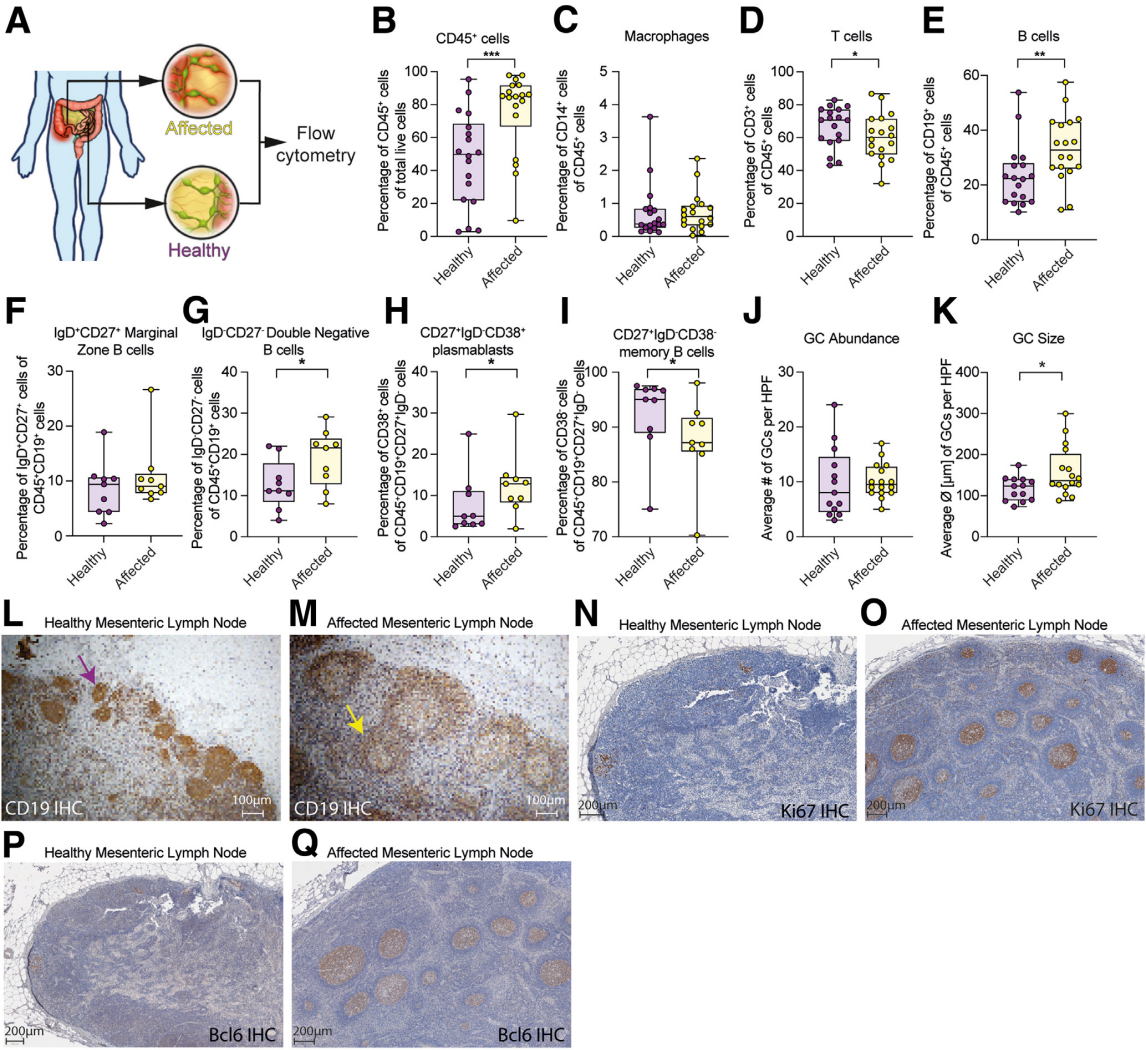
**B cells expand within affected draining MLNs.** (*A–I*) Characterization within paired draining MLNs of affected and healthy small intestinal segments. (*A*) Schematic depiction of sample collection. (*B–E*) Eighteen patients were included for basic immunologic phenotyping for (*B*) CD45^+^ cells, (*C*) CD45^+^CD14^+^ cells, (*D*) CD45^+^CD3^+^ cells, and (*E*) CD45^+^CD19^+^ cells. (*F–I*) A more detailed B-cell subset analysis of 9 patients showing (*F*) IgD^+^CD27^+^ marginal zone B cells, (*G*) IgD^-^CD27^-^ double-negative B cells of CD45^+^CD19^+^ B cells, (*H*) CD38^+^ cells, and (*I*) CD38^-^ cells of CD45^+^CD19^+^CD27^+^IgD^-^ B cells. (*J* and *K*) Differences in GC (*J*) abundance and (*K*) size per high-power field (HPF). (*L–Q*) Immunohistochemistry (IHC) staining: representative image of a draining MLN of (*L*) healthy and (*M*) affected small intestine stained for CD19. *Purple arrow* and *yellow arrow* indicate GCs. MLN of a (*N*) healthy small intestine and (*O*) affected segment stained for Ki67. MLN of a (*P*) healthy small intestine and (*Q*) affected segment stained for Bcl6. (*B–K*) A paired Student *t* test was used for analysis. ∗*P* < .05, ∗∗*P* < .01, and ∗∗∗*P* < .001.

Recent studies have investigated the role of B-cell responses in ulcerative colitis, which exclusively affects the colon, whereas data in Crohn’s disease (CD), which mainly affects the terminal ileum, are insufficient.^1^, ^2^, ^3^, ^4^ Granuloma formation within the thickened, inflamed mesentery of patients with CD, however, is associated with significantly worse outcome,^5^, ^6^, ^7^ and microstructural analysis has suggested increased numbers of B cells in CD mesentery.^8^ Although a healthy orchestrated mucosal immune response involves B-cell maturation and development of IgA-secreting plasma cells directed toward more invasive strains of bacteria, anticommensal IgG can be found in patients with CD, which usually are absent in healthy controls.^9^, ^10^, ^11^ Although a single-cell sequencing study in therapy-refractory CD found an intestinal immune cell signature in inflamed areas that included IgG^+^ plasmablasts, rather than IgA^+^ plasmablasts,^12^ B-cell receptor (BCR) sequencing in CD has been limited to peripheral blood to date.^13^ Anticommensal IgG antibodies, however, can be transported across mucosal barriers by a neonatal fragmented crystallizable region (Fc) receptor and sustain inflammation in ulcerative colitis, with data lacking for CD.^1^, ^2^, ^3^^,^^14^, ^15^, ^16^, ^17^ To investigate the local B-cell response in CD we therefore characterized paired samples of draining mesenteric lymph nodes (MLNs) of affected and adjacent healthy small intestinal segments (Figure 1*A*, Supplementary Table 1, Supplementary Methods, and Supplementary Figure 1). Fractions of CD45^+^ leukocytes were higher in affected areas (Figure 1*B*), macrophages were negligeable (Figure 1*C*), T cells were reduced (Figure 1*D*), and CD19^+^ B cell fractions were expanded in affected MLNs (Figure 1*E*). Further characterization of CD45^+^CD19^+^ cells^18^ within MLNs showed that IgD^+^CD27^+^ marginal zone B-cell fractions^19^ were comparable between affected and healthy MLNs (Figure 1*F*), IgD^-^CD27^-^ double-negative B cells were more abundant within CD45^+^CD19^+^ B cells of affected MLNs (Figure 1*G*), CD38^+^ plasmablasts numbers were increased and CD38^-^ memory B cells were reduced within the CD45^+^CD19^+^CD27^+^IgD^-^ B-cell fraction (Figure 1*H* and *I*), overall suggesting ongoing antigenic stimulation within affected MLNs.^20^^,^^21^

B cells mature within germinal centers (GCs), which are specific microanatomic structures within secondary lymphoid organs such as MLNs.^22^ Immunohistochemistry staining for CD19 showed that GC abundance was unchanged between affected and healthy MLNs (Figure 1*J*), but GCs in affected areas were significantly larger (Figure 1*K*). In addition, MLNs from healthy areas displayed GCs that appeared to be immature (Figure 1*L*), whereas affected MLNs often showed GCs with dark and light zones (Figure 1*M*). Because B-cell isotype switching within GCs is a T-cell–dependent process,^23^^,^^24^ we next performed immunohistochemistry staining for Ki67, a marker indicating cell proliferation,^25^ and Bcl6, a master regulator for T-follicular helper cells and expressed in class-switching B cells.^23^ Ki67 and Bcl6 staining marked individual cells scattered within the MLNs, but the majority of healthy MLNs showed a lack of positive GCs (Figure 1*N* and *P*), whereas Ki67 and Bcl6 were highly positive within GCs of affected MLNs (Figure 1*O* and *Q*).

**Figure 2:**
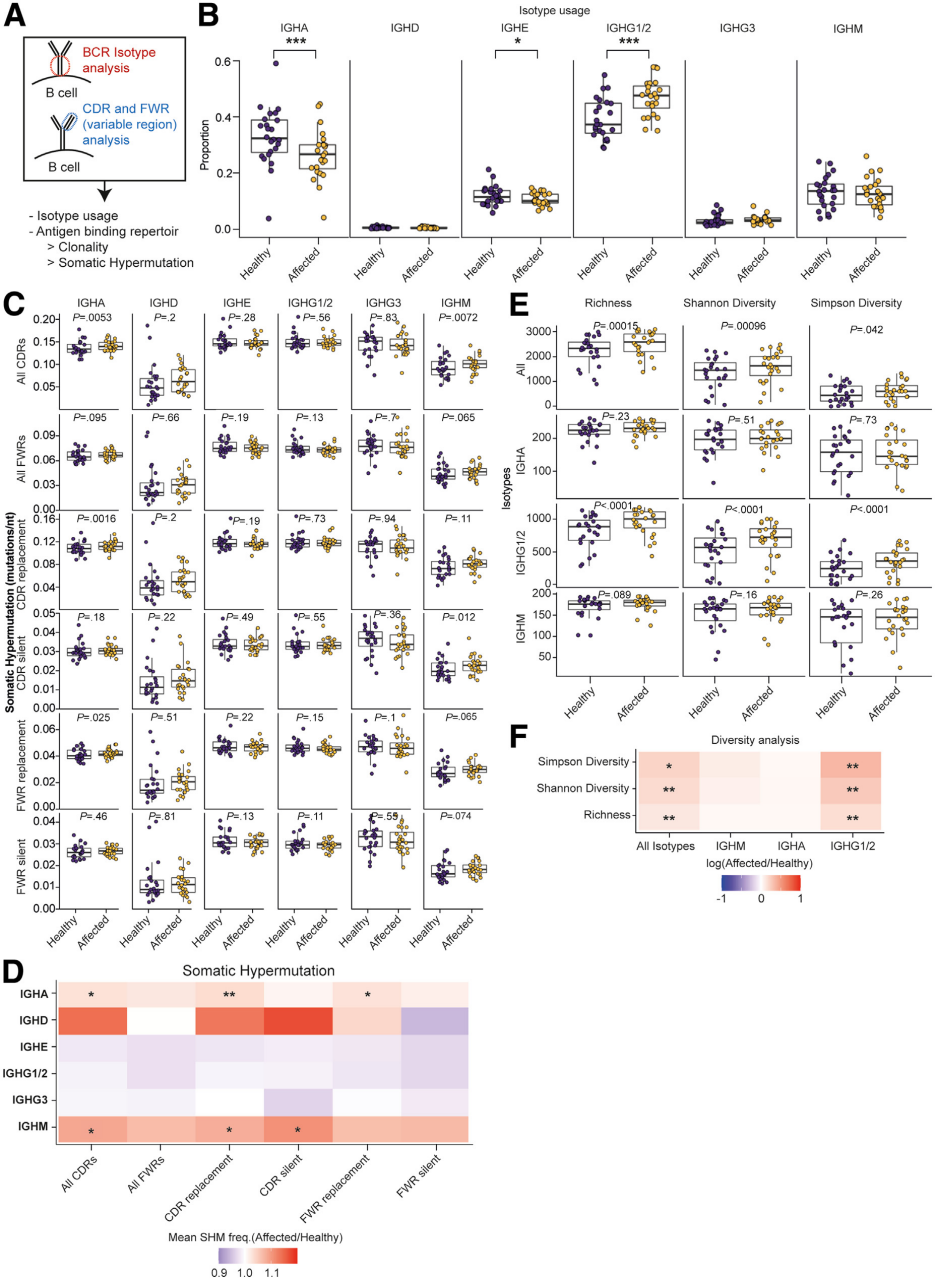
**B-cell–receptor sequencing indicates pathologic B-cell maturation.** (*A*) Schematic depiction of the BCR sequencing strategy performed in 24 patients. (*B*) BCR isotype use in MLNs. (*C*) Rate of SHM in CDRs or framework regions (FWRs). (*D*) Rate of SHM is depicted as the ratio of affected/healthy MLNs. (*E*) Diversity analysis on BCR sequences of the variable regions. Richness refers to the abundance of unique clones in a repertoire, Simpson’s index assesses the probability of 2 randomly sampled reads belonging to the same clone, Shannon’s index is a measure of evenness. (*F*) Depiction of individual results and (*E*) as log of affected/healthy. ∗*P* < .05, ∗∗*P* < .01, and ∗∗∗*P* < .001. freq., frequency; IGHA, Immunoglobulin Heavy Constant Alpha; IGHD, Immunoglobulin Heavy Constant Delta; IGHE, Immunoglobulin Heavy Constant Epsilon; IGHG, Immunoglobulin Heavy Constant Gamma; IGHM, Immunoglobulin Heavy Constant Mu.

Thus, BCR sequencing was performed to investigate differences in class switching between MLNs (Figure 2*A*) and showed decreased use of Immunoglobulin Heavy Constant Alpha (IGHA) and Immunoglobulin Heavy Constant Epsilon (IGHE), with a concomitant significant increase in Immunoglobulin Heavy Constant Gamma 1/2 (IGHG1/2) in affected MLNs (Figure 2*B*). Although isotype analysis can provide important information about class switching, analysis of the BCR variable region provides more information about affinity maturation. Affinity maturation, a process in which B cells increase their antigen affinity and avidity, occurs via somatic hypermutation (SHM) within the GC’s dark zone.^26^ Thus, mutation rates within complementary determining regions (CDRs) and framework regions (FWRs) of the BCR were compared and analyzed for silent mutations (ie, mutations that do not result in a change in amino acid sequences), and substitution mutations (ie, mutations that result in a change in amino acid sequence, and, therefore, Ig structure) (Figure 2*C*). The mean SHM frequency was significantly higher in IGHA as well as Immunoglobulin Heavy Constant Mu (IGHM) B cells (Figure 2*C* and *D*). This increased rate of SHM in affected MLNs was driven by replacement mutations in the CDRs as well as framework regions of IGHA B cells, and CDR mutations in IGHM B cells (Figure 2*C* and *D*). In line with this, diversity analysis indicated an increased diversity of the BCR in IGHG1/2 B cells whereas diversity was unchanged in IGHM or IGHA B cells (Figure 2*E* and *F*).

Overall, our results indicate ongoing class switching within draining MLNs of affected intestinal segments, with a shift toward IGHG1/2 BCRs. The lack of high SHM rates within IGHG1/2 BCRs, the difference between IGHA and IGHG1/2 BCRs in single MLNs, and increased diversity in IGHG1/2 BCRs suggests that many antigens do not result in long-lasting immunologic stimulation, and IGHA and IGHG1/2 responses may target different pathogens/commensals.
