# Supplementary material for: Altered B-Cell Expansion and Maturation in Draining Mesenteric Lymph Nodes of Inflamed Gut in Crohn’s Disease
Source: Cell Mol Gastroenterol Hepatol. 2023 Dec 24;17(4):662–6. doi: 10.1016/j.jcmgh.2023.12.006 (PMC10958343; doi:10.1016/j.jcmgh.2023.12.006)
Supplement: Supplementary Figure 1 [file mmc1.pdf]

SUPPLEMENTARY METHODS

METHODS

Patients

All samples analysed within the study were collected prospectively from patients undergoing surgery due to Crohn’s disease related complications requiring intestinal resections, or healthy volunteers. A total of 36 paired lymph node (LN) samples of 18 patients were used for basic flow cytometric analysis, and 18 paired samples of 9 patients were additionally analysed with an expanded B cell subset panel. A separate 48 paired samples of 24 patients undergoing surgery due to CD related complications was used for B cell receptor (BCR) sequencing (Figure 1, Supplementary Table 1). For immunohistochemical staining, spare LN samples that were not required for B cell receptor sequencing of a total of 14 patients were used, totalling 10 lymph nodes of affected regions and 10 lymph nodes of unaffected regions, respectively. The study was conducted in accordance with the Declaration of Helsinki and approved by the local institutional review board (EK #1480/2016 and #1915/2021) prior to study initiation. All patients included within the study gave their written informed consent prior to participation.

Lymph nodes flow cytometry

LN samples of the draining mesentery were collected of affected and unaffected intestinal segments of patients with Crohn´s disease (CD) undergoing surgery. All samples were directly taken after the specimen was resected within the operating theatre and put on ice. Samples were then transported to the laboratory, where they were processed to single cell suspensions and stained within 6 hours. During sample preparation and until fixing, all steps were carried out on ice. LN samples were processed to single cell suspensions and stained with flow cytometric antibodies to broadly characterize immune cells, as outlined below. LN tissue was cut with a sterile scissors in a petri-dish and pushed through a 70 µm cell strainer with washing buffer

(phosphate buffered saline (PBS), 2% fetal calf serum (FCS), 1mM EDTA) and the help of a syringe plunger to obtain a single cell suspension. Additional digestion with collagenase was omitted after pilot trials, as several of the respective epitopes were altered and could not be detected after collagenase digestion (data not shown). Cells were counted and $2x10^6$ cells per analysis were resuspended in 500 µl fixable viability stain 620 (BD Biosciences Cat# 564996, RRID:AB_2869636) and incubated in the dark for 15 minutes at 4°C. Thereafter, cells were washed in 500 µl of FACS buffer (PBS, 2% bovine serum albumin (BSA)), centrifuged (450 x g for 5 minutes at 4°C), resuspended in 50µl of Fc blocking solution (normal human serum (NHS) diluted 1:1 with FACS buffer), and incubated for 10 minutes at 4°C. Staining with the respective antibody mixes was performed for 20 minutes at 4°C, with the respective negative controls run alongside. The following mouse anti-human antibodies used within the study were obtained from BD Bioscience: CD123-BV421 (clone 9F5) (Cat# 562517, RRID:AB_11153668), CD3-BV510 (clone UCHT1) (Cat# 563109, RRID:AB_2732053), CD16-BV605 (clone 3G8) (Cat# 563172, RRID:AB_2744297), CD27-BB515 (clone M-T271) (Cat# 564642, RRID:AB_2744354), IgD-BB700 (clone IA6-2) (Cat# 566538, RRID:AB_2744486), CD14-PE-Cy7 (clone (M5E2) (Cat# 557742, RRID:AB_396848), CD56-APC (clone NCAM 16.2) (Cat# 341027, RRID:AB_2868759), CD19-APC-R700 (clone HIB19) (Cat# 564977, RRID:AB_2744308), CD11c-PE (clone S-HCL-3) (Cat# 333149, RRID:AB_2868650), CD45-APC-Cy7 (clone 2D1) (Cat# 348815, RRID:AB_2868859), and from Biolegend: CD24-BV605 (clone ML5) (Cat# 311123, RRID:AB_2562287), CD38-PE (clone HIT2) (Cat# 303506, RRID:AB_314358). After incubation, cells were washed twice with 500 µl FACS buffer, resuspended in 200 µl fixation buffer (FACS buffer with 0.25% formaldehyde) and stored at 4°C in the dark until measurement within the following 24 hours. The gating strategy is depicted in Supplementary Figure 1.

Data collection was performed on a BD LSRFortessa Flow Cytometer (RRID:SCR_019601), and data was analysed using Kaluza software (Beckman Coulter; RRID:SCR_016182). Statistical

analysis was performed with GraphPad Prism (version 9, GraphPad Software, LLC; RRID:SCR_002798). All respective antibodies and catalogue numbers are summarised in Suppl. Table 2.

Immunohistochemistry (IHC)

LN samples obtained for BCR sequencing that were big enough to be divided were divided and half of the samples was used for BCR analysis (see below), and the other half embedded in paraffin. A total of 10 samples for each group (affected and unaffected areas, respectively) were further used to investigate numbers and size of germinal centres.

Specimen were cut into 4µm histological sections and put at 60°C overnight. Deparaffinization and rehydration with a graded alcohol series starting from Xylene to 100%, 80%, 70%, 50% ethanol followed, and rehydration was finished with distilled water. To block endogenous peroxidase, slides were incubated for 10 minutes in the dark at room temperature in PBS with 0.3% hydrogen peroxide. After washing twice with PBS for 3 minutes, antigen retrieval was performed in 10mM Tri-sodium citrate buffer (pH 6.0) (Dako, Cat# S2369) in a steam autoclave (CertoClav) at 119°C. Once tissue was cooled to 70°C, slides were washed with PBS containing 0.1% Tween 20 (PBS-T) for 3 minutes, followed by incubation with normal goat serum 2.5% (Vector Laboratories, Cat# 30024) for 20 minutes at room temperature. Incubation of the primary antibody was performed with a 1:100 dilution of purified anti-human CD19, Ki67 or Bcl6 in PBS-T, containing 1% goat serum (Dako, Cat# X0907) at 4°C overnight. All respective used antibodies and catalogue numbers are summarised in Suppl. Table 2. Negative controls were done without primary antibody incubation. After washing twice with PBS-T for 3 minutes, slides were incubated with goat-anti-mouse IgG, conjugated with Horseradish Peroxidase (HRP) (Vector Laboratories, Cat# 30126) for 1 hour at room temperature, followed by two more washing steps with PBS-T for 3 minutes. Peroxidase labelling was visualized by incubation with 3,3´-diaminobenzidine (Liquid DAB+ Substrate Chromogen System, Dako, Cat# K3468). The reaction was stopped with distilled

water. Nuclei were counterstained with Gill´s hematoxylin (Merck, Cat# 1.05174.0500) for 2 minutes, and slides were collected in Scott´s bluing solution (Morphisto, Cat# 11192). After dehydration with a graded alcohol series, sections were collected in *n*-butyl acetate and mounted with Entellan (Merck, Cat# 1.07961.0100).

Quantification of the GC was performed per high power field (HPF). The diameter of GCs was determined using the ZEN Digital Imaging for Light Microscopy (Zeiss, RRID:SCR_013672). Statistical analysis was performed with GraphPad Prism (version 9, GraphPad Software, LLC; RRID:SCR_002798).

B cell receptor (BCR) sequencing

Snap frozen LN samples were obtained from surgical specimen of the draining mesentery of affected, and adjacent unaffected intestinal segments of 24 patients undergoing surgery for Crohn's disease related complications. RNA was extracted using RNeasy Midi Kits (Qiagen, Cat# 75144). For disruption and homogenization, tissue was cut into small pieces using a sterile scalpel and homogenized in Buffer RLT (Qiagen, Cat# 79216) containing β-mercaptoethanol using bead beating. Following the disruption step, the further RNA extraction was performed as per the manufacturer's instructions. RNA concentration was determined using a Thermo Scientific NanoDrop One/OneC Microvolume UV Vis Spectrophotometer (ThermoFisher; RRID:SCR_023005), and equal amounts of RNA were used for further analysis. BCR sequencing was performed as reported by Bashford-Rogers et al[13], and the detailed technical description of the used methodology can be found within the methods section of the respective manuscript. In brief, reverse transcription and amplification with barcoded primers was performed[13]. Sequencing libraries were then prepared as per the manufacturer's instructions (Illumina) and sequenced utilizing 250bp paired-end sequencing on a MiSeq (Illumina MiSeq System; RRID:SCR_016379).

BCR sequence data was processed using the Immcantation toolbox (v4.0.0). Raw sequence reads were filtered for base quality (median Phred score ≥32) using pRESTO. Forward and reverse reads were merged. Sequences were retained where over 80% base sequence similarity was present between all sequences with the same barcode. The constant-region allele with highest sequence similarity was identified by 10-mer matching to the reference constant-region genes from the IMGT database. Sequences without complete reading frames and non-immunoglobulin sequences were removed and only reads with significant similarity to reference IGHV and J genes from the IMGT database using BLAST were retained. Immunoglobulin variable gene use and sequence annotation were performed in IMGT V-QUEST.[e1]

Somatic hypermutation levels (including silent and non-silent mutations) per unique IGHV-D-J region per isotype were calculated over the CDR1 and CDR2 region for each individual sample using the observedMutation function within the SHazaM package.[e2] Identical sequences were clustered and each clone was only counted once. BCR clones were assigned using the Change-O package using the single-nucleotide Hamming distance model.[e2] The Alakazam package[e2] was used to analyse the BCR sequencing data for diversity estimation of CDR3 sequences, the diversity estimates were adjusted for sequencing depth via subsampling with 2,000 random iterations.[e3]

Supplementary References

e1. Brochet X, et al. Nucleic Acids Res. 2008;36:W503–W508.

e2. Gupta NT, et al. Bioinformatics. 2015;31:3356–3358.

e3. Shugay M., et al. PLoS Comput Biol. 2015;11:e1004503.

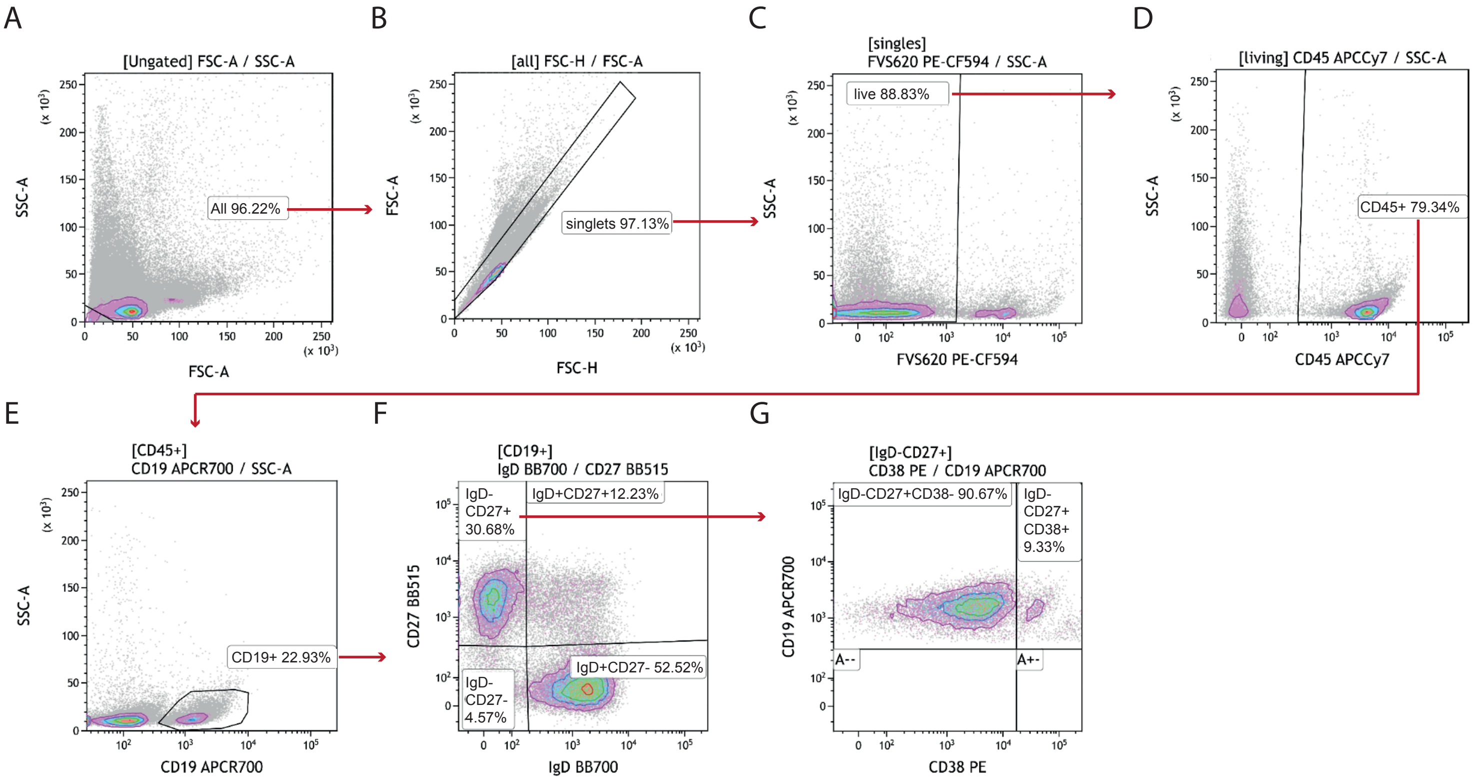

Supplementary Figure 1. Gating strategy for MLNs, depicted on a representative sample. (A) Forward and sideward scatter for all acquired cells. (B) Gating on single cells. (C) Exclusion of dead cells by utilising viability dye. (D) Gating on CD45+ cells. (E) Gating on CD19+ cells. (F) Gating on IgD-CD27+ cells. (G) Results for CD38 staining.

Supplementary Table 1. Clinical baseline characteristics at the time of surgery.

	Flow cytometry (n=18)	IHC (n=14)	BCR (n=24)
**Age [mean (±SD)]**	39.1 (±14.3)	40.2 (±15.8)	42.1 (±14.9)
**Gender**	Male = 7<br>Female = 10	Male = 8<br>Female = 6	Male = 14<br>Female = 10
**Smoker at the time of surgery**	Yes = 5<br>No = 13	Yes = 3<br>No = 11	Yes = 5<br>No = 19
**Preoperative CRP [mg/dL; mean (±SD)]**	1.9 (±1.9)	3.9 (±4.6)	3.5 (±4.6)
**Pre-operative therapy**	TNFi = 4<br>AZA = 0<br>Mesalazine = 2<br>Ustekinumab = 2<br>Vedolizumab = 2<br>None = 8	TNFi = 3<br>AZA = 4<br>Mesalazine = 0<br>Ustekinumab = 0<br>Vedolizumab = 0<br>None = 7	TNFi = 7<br>AZA = 4<br>Mesalazine = 0<br>Ustekinumab = 1<br>Vedolizumab = 1<br>None = 11
**Disease location[+]**	Ileocolonic = 14<br>Colonic = 2<br>Small intestine = 2	Ileocolonic = 7<br>Colonic = 1<br>Small intestine = 6	Ileocolonic = 12<br>Colonic = 2<br>Small intestine = 10
**Histology**	Stenosis = 10<br>Ulceration = 15<br>Fistula = 7<br>Peritonitis = 6<br>Abscess = 1	Stenosis = 6<br>Ulceration = 12<br>Fistula = 2<br>Peritonitis = 4<br>Abscess = 3	Stenosis = 11<br>Ulceration= 16<br>Fistula = 4<br>Peritonitis = 3<br>Abscess = 5
**Perianal disease**	Yes = 8<br>No = 10	Yes = 4<br>No = 10	Yes = 8<br>No = 16

Abbreviations: AZA Azathioprine; BCR B cell receptor sequencing; CRP C reactive protein; IHC Immunohistochemistry; TNFi TNF-alpha inhibitor

Supplementary Table 2. Used antibodies for flow cytometry and IHC.

Antibodies	Source	Identifier
Flow cytometry		
Mouse anti-human CD123-BV421 (clone 9F5)	BD Bioscience	Cat# 562517, RRID:AB_11153668
Mouse anti-human CD3-BV510 (clone UCHT1)	BD Bioscience	Cat# 563109, RRID:AB_2732053
Mouse anti-human CD16-BV605 (clone 3G8)	BD Bioscience	Cat# 563172, RRID:AB_2744297
Mouse anti-human CD27-BB515 (clone M-T271)	BD Bioscience	Cat# 564642, RRID:AB_2744354
Mouse anti-human IgD-BB700 (clone IA6-2)	BD Bioscience	Cat# 566538, RRID:AB_2744486
Mouse anti-human CD14-PE-Cy7 (clone (M5E2)	BD Bioscience	Cat# 557742, RRID:AB_396848
Mouse anti-human CD56-APC (clone NCAM 16.2)	BD Bioscience	Cat# 341027, RRID:AB_2868759
Mouse anti-human CD19-APC-R700 (clone HIB19)	BD Bioscience	Cat# 564977, RRID:AB_2744308
Mouse anti-human CD11c-PE (clone S-HCL-3)	BD Bioscience	Cat# 333149, RRID:AB_2868650
Mouse anti-human CD45-APC-Cy7 (clone 2D1)	BD Bioscience	Cat# 348815, RRID:AB_2868859
Mouse anti-human CD24-BV605 (clone ML5)	Biolegend	Cat# 311123, RRID:AB_2562287
Mouse anti-human CD38-PE (clone HIT2)	Biolegend	Cat# 303506, RRID:AB_314358
Immunohistochemistry		
Monoclonal mouse anti-human CD19 (clone A17136C)	Biolegend	Cat# 396302, RRID:AB_2894554
Monoclonal mouse anti-human Ki67 (clone MIB-1)	Agilent	Cat# M7240, RRID:AB_2142367
Monoclonal mouse anti-human Bcl6 (clone PG-B6p)	Agilent	Cat# M7211, RRID:AB_2063451
